# Appraisal of the New Posture Analyzing and Virtual Reconstruction Device (PAViR) for Assessing Sagittal Posture Parameters: A Prospective Observational Study

**DOI:** 10.3390/ijerph191711109

**Published:** 2022-09-05

**Authors:** Chan Woong Jang, Jihyun Park, Han Eol Cho, Jung Hyun Park

**Affiliations:** 1 Department of Rehabilitation Medicine, Gangnam Severance Hospital, Rehabilitation Institute of Neuromuscular Disease, Yonsei University College of Medicine, Seoul 06229, Korea; 2 Department of Rehabilitation Medicine, Hallym University Dongtan Sacred Heart Hospital, Hallym University College of Medicine, Hwaseong 18450, Korea; 3 Department of Medical Device Engineering and Management, Yonsei University College of Medicine, Seoul 06229, Korea

**Keywords:** diagnostic imaging, imaging, three-dimensional, posture, skeleton, spine

## Abstract

The purpose of this study was to report the clinical validation of the posture analyzing and virtual reconstruction device (PAViR) system, focusing on the accuracy of sagittal spinal parameters, compared with the EOS imaging system. Seventy patients diagnosed with segmental and somatic dysfunction were recruited between February 2020 and November 2020. Each patient was examined using the EOS imaging system and PAViR; the sagittal parameters of human body posture [forward head posture (FHP), T1 tilt angle (T1t), knee flexion angle (KF), lumbar lordosis angle (LL), and pelvic tilt angle (PT)] were analyzed to verify the correlation between the results of the two devices. The median differences in the results of the two devices showed significant differences in FHP (T4-frontal head and T4-auditory canal), T1t, and PT. In the correlation analysis, the values of FHP (C7-auditory canal, T4-frontal head, and T4-auditory canal), T1t, and PT showed a moderate correlation between the two devices (r = 0.741, 0.795, 0.761, 0.621, and 0.692, respectively) (*p* < 0.001). The KF and LL was fairly correlated (r = 0.514 and 0.536, respectively) (*p* = 0.004, both). This study presents the potential of a novel skeletal imaging system without radiation exposure, based on a 3D red-green-blue-depth camera (PAViR), as a next-generation diagnostic tool by estimating more accurate parameters through continuous multi-data-based upgrades with artificial intelligence technology.

## 1. Introduction

Posture, defined as the alignment or orientation of the body in an upright position, is related to the ability to perform effective energy-conserving movements and to protect the body structure from injury or progressive deformity [1,2]. Musculoskeletal abnormalities are the main cause of most postural changes. In particular, an abnormal posture of the sagittal spine represents an imbalance of the trunk and pelvis [3,4]. A precise and reliable evaluation of the posture is essential for the clinician’s planning and decision-making regarding the management of musculoskeletal pain.

There are several methods for assessing posture based on radiographic and non-radiographic measurements [5,6,7,8]. Currently, radiography is the gold standard for evaluating posture; a radiographic method with high accuracy is the EOS imaging system (Biospace Med, Paris, France), which is a low-dose, whole-body medical radiographic diagnostic device. High-quality images have been created using the EOS imaging system with technological updates and advances that provide diagnostic relevance to abnormalities and deformities of the spine, pelvis, and lower extremities via numerous criteria [9,10,11]. However, this method has some disadvantages. First, it is unaffordable in general clinical practice because of the high maintenance and staffing costs. Second, radiographs obtained using X-ray radiation, including the EOS imaging system, are still invasive and hazardous. A previous study found that in comparison to conventional radiography, the total radiation dose used by the EOS imaging system was reduced by approximately 50% [12]. This has led to an emerging interest in low-cost, non-invasive methods of measuring posture without radiation risk [13].

Advancements in sensors, processing units, and machine learning algorithms have led to the emergence of real-time sensor-based technologies that detect human body posture, such as three-dimensional (3D) depth cameras. Thus, new posture analysis and virtual reconstruction device (PAViR), which uses a real-time 3D red-green-blue-depth (RGB-D) camera, was developed. Through PAViR, the human posture can be evaluated more quickly with no radiation. However, there is a lack of information on diagnostic eligibility, particularly for the sagittal parameters of PAViR. Therefore, the purpose of this study determines whether PAViR is reliable when used as a diagnostic imaging tool in comparison to the sagittal parameters of the EOS imaging system.

## 2. Materials and Methods

### 2.1. Participants

Patients diagnosed with segmental and somatic dysfunction (M99.0) according to the International Classification of Diseases, Tenth Revision, and confirmed spinal pathologies in the Department of Rehabilitation Medicine at a tertiary hospital were recruited from February 2020 to November 2020. Segmental and somatic dysfunction are aggravated by poor posture or results in an abnormal posture that leads to dysfunctional mechanics. Dysfunction is the result of a complicated interplay of a whole chain of linked structures. Thus, it would be more helpful to understand a patient’s condition if the whole body were imaged rather than a part of the body. Therefore, subjects with segmental and somatic dysfunction are suitable to evaluate the correlation between the results of the EOS imaging system and PAViR.

The exclusion criteria were as follows: (1) those who were diagnosed with other neurological or orthopedic pathologies, (2) those who were younger than 19 years of age, (3) those with a body mass index (BMI) ≥ 35 kg/m^2^ or higher, (4) those who had a metallic fixation device inserted after spinal surgery, and (5) those who could be or were pregnant. Based on the findings of a comparative study, a sample size of 40 participants was selected to provide a statistical power of up to 80% and an alpha error of 5% [14].

This study was approved by the Institutional Review Board of Gangnam Severance Hospital, Seoul, Republic of Korea (Identifier: 3-2019-0303), and informed consent was obtained from all the study participants. The study protocol was conducted in accordance with the tenets of the Declaration of Helsinki.

### 2.2. Data Assessment

#### 2.2.1. Posture Assessment

In this prospective observational study, each participant was examined as part of a routine clinical protocol during their initial clinical visit. When taking the EOS imaging or full-body standing orthogonal anteroposterior and lateral X-ray, individuals were instructed to stand in the modified standing stance with their shoulders flexed to 45° and their fists placed on their clavicles for sagittal visualization of the cervical and thoracic spine regions [15,16]. Then, conventional angle measurements were performed manually on a digital lateral EOS image by one skillful examiner.

On the same day, PAViR (version 2.11, Moti Physio, MG Solutions, Seoul, Korea) measurements for each patient were also performed using a 3D RGB-D camera (Astra Pro, Orbbec 3D Technology International, Inc., Troy, MI, USA) as a sensor. Individuals were evaluated in a standing position with crossed arms and the head looking forward. This position, which is not generally recommended for PAViR, was adopted to facilitate comparison with the EOS imaging measurements. The patients were fully dressed in relatively tight clothes with chest and waistbands to make the silhouette visible. Markers were manually placed at the level of the right and left anterior superior ischial spine and umbilicus, respectively. Anatomical landmarks were pointed on the virtual skeletal model, and values were measured by a physical medicine and rehabilitation specialist under close double-checking by a second specialist.

#### 2.2.2. PAViR

The hardware system of PAViR consists of a display, input, operation, and positioning unit. The display unit provides visual feedback on the correct protocol positioning and information on the final estimation result. The input unit, the 3D RGB-D camera, receives the depth and the points (acromial end, anterior superior ischial spine, center of thigh, knee, and ankle on the front view; external auditory meatus, lateral aspect of greater tubercle of shoulder, center of pelvis and knee, lateral malleolus on the lateral view, acromion on the back view) to be measured with 30,000 reflected beams as input data. The operation unit processes the input data to calculate the image source for graphical data and displays it on the display unit. The positioning unit consisted of a laser indicator, lighting on the floor at a defined distance from the 3D RGB-D camera, and a floor mat to stand on a specific area.

**Figure 1:**
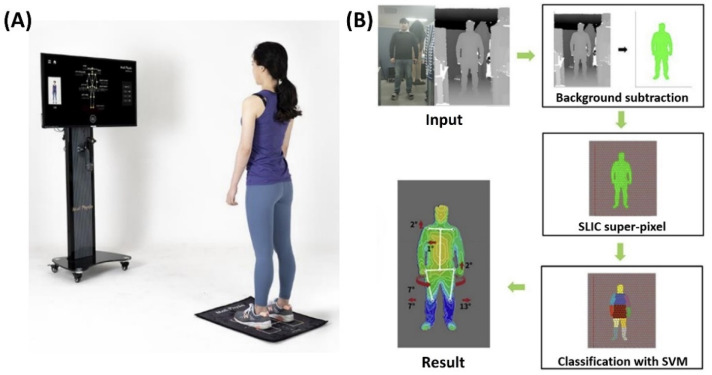
(**A**) Posture analyzing and virtual reconstruction device (PAViR) system. (**B**) The process of reconstructing human posture measurements using PAViR. SLIC, simple linear iterative clustering; SVM, support vector machine.

The 3D RGB-D camera captures images from the front, lateral, and back. The system generates an outline of the human silhouette from the depth values of the images using the background-subtracted extraction method [17]. The body parts of the subject are identified using a support vector machine, such as the head, neck, trunk, arms, and legs, using an image processing algorithm for super-pixel segmentation known as simple linear iterative clustering [18,19]. This process is calculated for the image frames every 2–3 s, and the angle estimated for each joint is used as the value for the final result based on the average joint position and applied to the 3D virtual skeleton model. The results, or the posture, were indirectly presented on the screen as an automatically processed virtual skeletal model with coronal and sagittal images through repeated training sessions for the human pose estimation algorithm (Figure 1) [18,20].

#### 2.2.3. Outcome Measures

**Figure 2:**
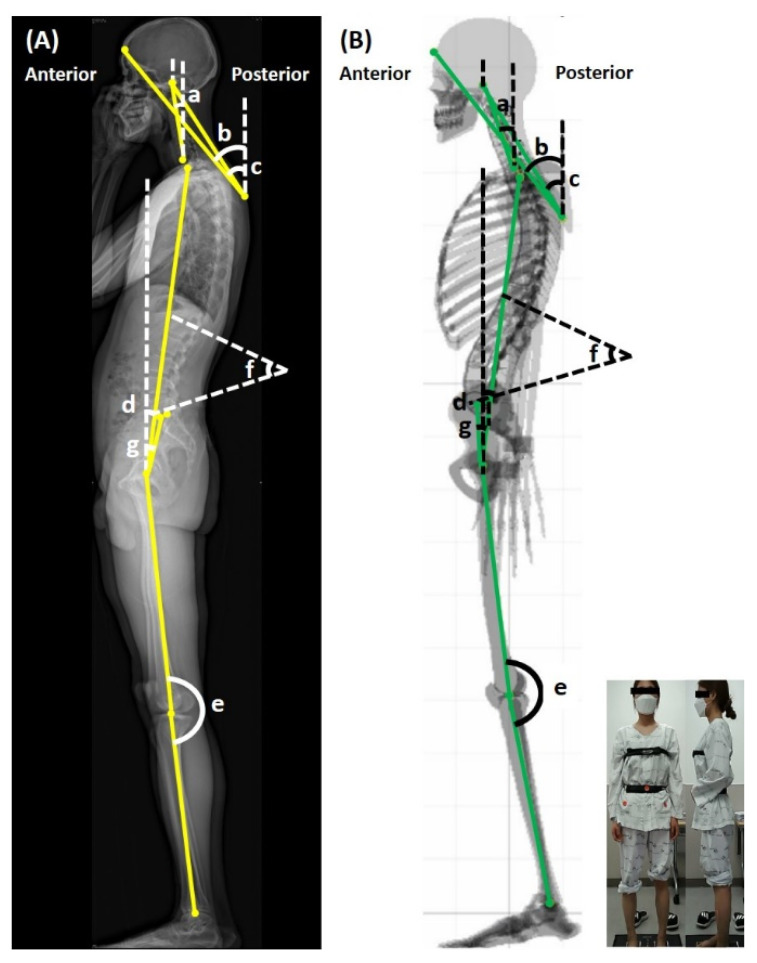
Methods of measuring various sagittal parameters of (**A**) the EOS imaging system and (**B**) PAViR. a, forward head posture (C7-center of auditory canal); b, forward head posture (T4-frontal head); c, forward head posture (T4-center of auditory canal); d, T1 tilt angle; e, knee flexion angle; f, lumbar lordosis angle; g, pelvic tilt angle.

The primary outcomes were the following sagittal parameters of human body posture obtained by the EOS imaging system and PAViR: (1) forward head posture (FHP) in three different methods (from the center of the acoustic meatus to the center of the C7, from the back at the T4 level to the frontal head, and from the back at the T4 level to the center of the acoustic meatus); (2) T1 tilt angle (T1t) formed by the vertical axis traversing the center of the femoral head; (3) knee flexion angle (KF) by the axis joining the center of the hip to the center of the knee and the axis joining the center of the knee to the center of the ankle; (4) lumbar lordosis angle (LL) formed between the line extending from the upper plate of the L1 and the other extending from the lower plate of the L5; and (5) pelvic tilt angle (PT) formed between the vertical axis traversing the center of the femoral head and the midpoint of the sacral endplate (Table 1 and Figure 2). These were analyzed to verify the correlation between the results of the EOS imaging and PAViR for validation.

### 2.3. Data Analysis

Descriptive data are shown as the median (interquartile range [IQR]) or number (percentage, %). The test for normality for continuous variables was performed using the Shapiro–Wilk normality test. Accuracy was assessed using the Wilcoxon signed-rank test of absolute mean differences and *p*-values between the results of the EOS imaging and PAViR. Associations between measurements of the two devices were determined using Spearman’s rank correlation coefficients, which were interpreted as none (0.0), poor (0.01–0.29), fair (0.30–0.59), moderate (0.60–0.79), very strong (0.80–0.99), and perfect (1.0) [21]. The level of significance was set at *p* < 0.05 for all statistical tests. All analyses were performed using R Studio software (R version 4.1.2; RStudio, PBC, Bonston, MA, USA).

## 3. Results

### 3.1. Participants

Seventy patients were included in the study. There were 30 men and 40 women with a median age of 55 years (range, 23–82 years), median height of 165 (range, 147–185 cm), median weight of 65 (range, 43–100 kg), and median body mass index of 23.62 kg/m^2^ (ranging, 16.38 to 31.25) (Table 2).

### 3.2. Descriptive Outcome Values

The descriptive values of sagittal parameters measured using the EOS imaging system and PAViR are presented in Table 3. Negative values represent posteriorly tilted status. The outcomes of FHP (C7-auditory canal, T4-frontal head, and T4-auditory canal) measured by the EOS imaging system showed similar median values and IQR to those estimated by PAViR. The median values of T1t by the EOS imaging system and PAViR were 4.75° (2.83–6.48) and 2.95° (2.01–4.60), respectively. The median values of KF were 2.50 (1.23–5.28) and 3.90 (2.02–5.38) for the EOS imaging system and PAViR, 34.00 (29.00–42.00) and 34.55 (31.10–38.65) for the LL, and 12.55 (6.53–17.93) and 9.35 (6.05–10.48) for the PT, respectively.

### 3.3. Validity of PAViR Measurement

As summarized in Table 4, the median differences in the results of measurements by the two devices showed significant differences in FHP (T4-frontal head and T4-auditory canal), T1t, and PT. There were no significant differences in the other parameters.

In the correlation analysis, the values of the FHP of all three methods (C7-auditory canal, T4-frontal head, and T4-auditory canal), T1t, and PT estimated by PAViR showed a moderate correlation, with Spearman correlation coefficients of r = 0.741, 0.795, 0.761, 0.621, and 0.692, respectively (*p* < 0.001). The KF and LL estimated using PAViR were fairly correlated with that estimated using the EOS imaging system with statistical significance (r = 0.514 and 0.536, *p* = 0.004).

## 4. Discussion

The aim of this study was to evaluate the validity of a newly developed posture analysis and virtual reconstruction device (PAViR) to assess sagittal spinal parameters in patients with segmental and somatic dysfunction. The FHP using three different methods, T1t, KF, LL, and PT of PAViR were compared with those of the EOS imaging system. Our results indicate that PAViR can assess patients’ sagittal spinal parameters with a fair to moderate correlation to the EOS imaging system and has the potential to be a diagnostic tool with the need to increase accuracy.

Sagittal spinal misalignment severity is a key factor in musculoskeletal pain [22,23]. Traditional non-radiographic methods, including physical examination based on inspection and palpation for assessing postural asymmetry, were found to be very subjective and had several limitations [24,25,26,27,28]. Thus, efforts have been made to develop tools that can provide precise and regular postural alignment feedback. The EOS imaging system is one of the most objective and accurate methods for diagnosing posture among the devices that have been released so far [10,29]. However, despite its accuracy, several limitations of regular usage exist [30,31].

In this respect, PAViR has several benefits over the EOS imaging system: (1) no risk of additional radiation even if it is performed repeatedly, (2) relatively low cost, (3) no complicated installation, (4) fewer space constraints, and (5) easy system maintenance. Additionally, although the EOS imaging system requires an inpatient visit to the installation site and at least one physician or imaging technician for accurate posture and measurements, PAViR represents a direction for future development in that it can be implemented with equipment equivalent to a 3D RGB-D camera (e.g., a mobile phone or computer with a camera). Thus, it can be used as a tool for a home-based digital care system in the future without installation limitations or automated systems. Because of these advantages, methods using a 3D depth camera as a tool to evaluate human posture and skeletal imbalance will become a new technology that can replace existing spinal deformity evaluation tools.

Although the correlation between the two devices was somewhat good, there was a significant difference in some parameters, suggesting that there is room for improvement. First, it is important to minimize the errors caused by the patient’s examination clothing. We attempted to reduce the error by wearing chest and waistbands and attaching three markers; however, the condition of wearing clothes can cause distortion in surface measurements and topographic data. In particular, when patients wear black clothes, topographical data are inaccurate and indistinguishable from the background. To clarify the validity, further study will be necessary to be investigated with analyses with different clothing or on undressed patients. Second, it was necessary to reduce the impact of the test position. PAViR estimates the skeletal landmarks while patients move naturally. It measures the patient’s side with both arms lowered or gathered in front of the chest or abdomen and the front and back in a natural standing position with both arms lowered. However, the examination posture in the EOS imaging system does not exactly match that in PAViR. In this study, only patients who gathered in front of the chest posture were included to reduce the resulting error; however, the authors thought that this effect caused a significant difference in the test values. Lastly, the EOS imaging system displays the real curvatures of the whole spine, which is why spine surgeons use it for spinal correction surgery, whereas PAViR does not. This now limits the scope of use of PAViR for physical therapists and general practitioners.

However, in the future, PAViR could increase the accuracy of massive datasets based on machine learning technology, even when patients are wearing clothes or in different postures. Because it is possible to analyze the position of the neck, shoulder, pelvis, and knee by performing human posture estimation from the shape of the whole body, the potential for increasing the value of PAViR as a future diagnostic tool is limitless.

PAViR can acquire data that objectively show human posture by analyzing the image viewed from the outside without radiation using a 3D RGB-D camera. In this study, the values measured using PAViR were compared with the sagittal parameters obtained by performing the EOS imaging system in patients with segmental and somatic dysfunction. Among the results of PAViR, the values indicating FHP, T1t, KF, LL, and PT showed a statistically significant, moderate, and fair correlation, indicating validity compared with that of EOS imaging. Currently, the latest update of PAViR is version 2.13. Future research should proceed in the direction of suggesting and verifying the update, and it will be necessary to check whether the correlation we found increases accordingly.

## 5. Conclusions

This study presents the potential of a novel skeletal imaging system without the risk of radiation exposure, based on a 3D RGB-D camera, as a next-generation diagnostic tool by estimating more accurate parameters through continuous multi-data-based upgrades with artificial intelligence technology. PAViR provides acceptable validation compared to the EOS imaging system in sagittal human posture and may be useful as an alternative to EOS imaging for evaluating sagittal imbalance.

## Figures and Tables

**Table 1:** Description of the sagittal parameters of interest.

Parameter	Parameter’ Full Name	Description
FHP (C7-CAC), °	Forward head posture (C7-center of auditory canal), °	Angle formed between the center of the 7th cervical vertebra (C7) to the center of the auditory canal
FHP (T4-FH), °	Forward head posture (T4-frontal head), °	Angle formed between the back at the 4th thoracic vertebra (T4) to the frontal head
FHP (T4-CAC), °	Forward head posture (T4-center of auditory canal), °	Angle formed between the back at the 4th thoracic vertebra (T4) to the center of the auditory canal
T1t, °	T1 tilt angle, °	Angle formed between the center of the 1st thoracic vertebra (T1) and the vertical axis traversing the center of the femoral head
KF, °	Knee flexion angle, °	Angle formed by the axis joining the center of the hip joint to the center of the knee joint and the axis joining the center of the knee joint to the center of the ankle joint
LL, °	Lumbar lordosis angle, °	Angle formed between the line extending from the upper plate of the 1st lumbar vertebra (L1) and the other extending from the lower plate of the 5th lumbar vertebra (L5)
PT, °	Pelvic tilt angle, °	Angle formed between the vertical axis traversing the center of the femoral head and the midpoint of the sacral endplate

FHP (C7-CAC), forward head posture (C7-center of the auditory canal); FHP (T4-CAC), forward head posture (T4-center of auditory canal); FHP (T4-FH), forward head posture (T4-frontal head); KF: knee flexion angle; LL: lumbar lordosis angle; PAViR: posture analysis and virtual reconstruction device; PT: pelvic tilt angle; T1t: T1 tilt angle; °: degree of angle.

**Table 2:** Patient demographic and anthropometric characteristics (*n* = 70).

Characteristics	Median (IQR)	Range (Min–Max)
Sex, *n* (%)		
Male	30 (42.86)	
Female	40 (57.14)	
Age, yr	55 (38–63)	23–82
Height, cm	165 (159–172)	147–185
Weight, kg	65 (56.25–73.00)	43–100
BMI, kg/m^2^	23.62 (21.69–25.33)	16.38–31.25

BMI, body mass index; IQR, interquartile range.

**Table 3:** Descriptive values of the sagittal parameters measured with EOS imaging system and PAViR.

Sagittal Parameters	EOS Imaging System		PAViR	
	Median (IQR)	Range (Min–Max)	Median (IQR)	Range (Min–Max)
FHP (C7-CAC), °	8.80 (5.13–15.50)	−2.10–30.80	10.24 (6.35–14.47)	−6.30–29.00
FHP (T4-FH), °	41.40 (39.43–43.73)	32.00–54.20	40.54 (39.08–41.90)	33.90–47.00
FHP (T4-CAC), °	31.60 (28.25–34.80)	20.40–45.10	32.85 (30.23–35.10)	21.80–54.30
T1t, °	4.75 (2.83–6.48)	−5.70–11.30	2.95 (2.01–4.60)	−0.83–12.50
KF, °	2.50 (1.23–5.28)	0–18.90	3.90 (2.02–5.38)	−4.05–13.80
LL, °	34.00 (29.00–42.00)	7.00–61.00	34.55 (31.10–38.65)	23.00–45.80
PT, °	12.55 (6.53–17.93)	−2.50–35.00	9.35 (6.05–10.48)	0.70–22.40

FHP (C7-CAC), forward head posture (C7-center of the auditory canal); FHP (T4-CAC), forward head posture (T4-center of auditory canal); FHP (T4-FH), forward head posture (T4-frontal head); KF: knee flexion angle; LL: lumbar lordosis angle; PAViR: posture analysis and virtual reconstruction device; PT: pelvic tilt angle; T1t: T1 tilt angle; °: degree of angle.

**Table 4:** Accuracy and correlation of sagittal parameters measurements between EOS imaging system and PAViR.

Sagittal Parameters	Wilcoxon Signed Rank Test		Spearman’s Correlation	
	Median (IQR) of Difference	*p*-Value	Correlation Coefficient	*p*-Value
FHP (C7-CAC), °	1.87 (0.83–4.38)	0.418	0.741	<0.001
FHP (T4-FH), °	1.90 (0.86–3.05)	0.034	0.795	<0.001
FHP (T4-CAC), °	1.84 (0.78–3.05)	<0.001	0.761	<0.001
T1t, °	1.50 (0.67–2.67)	0.017	0.621	<0.001
KF, °	1.40 (0.80–3.35)	0.207	0.514	0.004
LL, °	4.05 (1.63–7.28)	0.701	0.536	0.004
PT, °	3.05 (1.23–6.58)	0.046	0.692	<0.001

FHP (C7-CAC), forward head posture (C7-center of the auditory canal); FHP (T4-CAC), forward head posture (T4-center of auditory canal); FHP (T4-FH), forward head posture (T4-frontal head); KF: knee flexion angle; LL: lumbar lordosis angle; PAViR: posture analysis and virtual reconstruction device; PT: pelvic tilt angle; T1t: T1 tilt angle; °: degree of angle.

## Data Availability

The aggregated data analyzed in this study are available from the corresponding author upon reasonable request.
